# Perforation of Meckel’s diverticulum by a wood splinter in a 4-year-old child: a case report

**Published:** 2013-06-25

**Authors:** D Anyfantakis, M Kastanakis, A Papadomichelakis, I Kokinos, N Katsougris, G Petrakis, P Karona, E Bobolakis

**Affiliations:** * Primary Health Care Centre of Kissamos, Chania, Crete, Greece; ** First Surgery Department, Saint George General Hospital, Chania, Crete, Greece

**Keywords:** Meckel’s diverticulum, perforation, foreign body, diagnosis

## Abstract

Perforation of Meckel's diverticulum by a foreign body represents an unusual and serious clinical occurrence. We present a case of a 4-year-old male who was admitted with symptoms of abdominal pain in the right iliac fossa, raising the suspicion of acute appendicitis. Exploratory laparotomy disclosed normal appendix and perforation of Meckel's diverticulum caused by a wood splinter. Meckel's diverticulectomy was performed and the child had an uneventful postoperative course. Preoperative diagnosis of perforated Meckel’s diverticulum remains a challenging issue. Diagnosis should be considered in the presence of a right lower quadrant abdominal pain or a positive history of ingestion of a sharp foreign body

## Introduction

Meckel’s diverticulum represents the most frequent congenital anomaly of the gastrointestinal tract with a reported prevalence of approximately 2% in the general population [**1**]. Regarding the gender, although it occurs equally in both sexes, complications appear more frequently among males, with a reported three to four times greater incidence in males [**1**]. It is formed by incomplete obliteration of the omphalomesenteric duct and is located on the antimesenteric border of the terminal ileum [**1**]. The structure is usually regressed between the fifth and seventh week of the fetal life [**1**]. Meckel’s diverticulum contains all three intestinal layers and maintains a separate blood supply from the viteline artery [**1**]. It is usually asymptomatic in the majority of cases for life [**1**]. We present an unusual case of perforation of Meckel’s diverticulum in a 4-year-old child caused by a wood splinter. 

## Case report

**Fig. 1:**
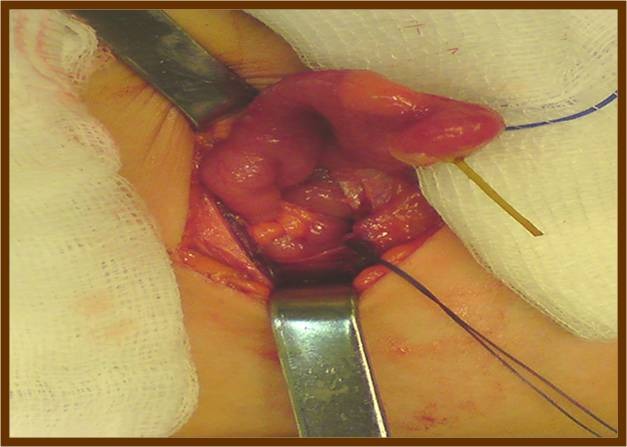
Inflamed and perforated Meckel’s diverticulum by a wood splinter

A previously healthy 4-year-old male was admitted to the emergency department of our institution via his general practitioner, due to a 24h history of abdominal pain, anorexia, nausea and two episodes of vomiting. Physical examination disclosed rebound abdominal tenderness located in the right lower quadrant, below and to the right of the umbilicus. Intestinal sounds were normal. His vital signs on admission were as it follows: blood pressure, 110/60 mmHg; pulse, 97 beats per minute; temperature, 37.1o Celsius; oxygen saturation 98% while breathing ambient air. Initial laboratory work up revealed normal findings except for leukocytosis (11.5 x 103/μL) and elevated C-reactive protein levels (3.2 mg/dl). Erect chest and supine abdominal radiographs were normal. A presumed diagnosis of acute appendicitis was made and the child underwent urgent exploratory laparotomy. Inspection of the intestinal loops disclosed a normal appendix. On further examination of the small bowel, an inflamed Meckel’s diverticulum was discovered perforated by a wood splinter which has protruded outside the diverticulum (**Fig. 1**). 

 Appendicectomy and resection of the perforated Meckel’s diverticulum were performed. The patient’s clinical recovery was uneventful and was discharged on the seventh postoperative day. 

## Discussion

Meckel’s diverticulum was initially described by Fabricius Hildanus in 1598 and named by Johann Friedrich Meckel who established its embryonical source of origin in a cadaver study of 22 children in 1809 [**2**]. In our case, the child presented with symptoms of acute abdomen. Definitive diagnosis and management required exploratory laparotomy, which disclosed a perforated Meckel’s diverticulum by a wood splinter that had been swallowed accidentally. 

 Incidence rate of complications due to Meckel’s diverticulum range from 4-16% [**1**]. Haemorrhage is the most frequent complication of the condition in the paediatric population and arises secondary to ulceration caused by acid secretion from ectopic mucosa [**1**]. Obstruction due to intussusception or adhesions, ulceration and inflammation (diverticulitis) are complications occurring more frequently among adults [**1**]. Remarkably, in a study among 71 pediatric patients diagnosed with Meckel’s diverticulum, painless rectal bleeding was the most prevalent initial manifestation [**3**]. 

 Perforation of Meckel’s diverticulum by a foreign body represents a rare complication [**1**] since the majority of the ingested objects pass through the gastrointestinal tract without problem [**4**]. Concerning the mechanism of perforation, it has been suggested to be the result of a combined effect of a local bacterial inflammation along with stasis and pressure necrosis to the diverticular wall [**5**]. In the majority of cases, the patients do not recall the ingestion. Perforation of Meckel’s diverticulum by a wood splinter has been rarely reported in literature [**6**]. Fish bones, chicken bones [**4**] and food items are the most common causes of Meckel’s diverticulum perforation [**7**]. 

 Abdominal tenderness located in the right lower quadrant along with leucocytosis and moderate elevated temperature, are the main clinical features that consist of the initial presentation of a perforated Meckel’s diverticulum [**6**]. Because clinical, laboratory and imaging features of complicated Meckel’s diverticulum are not pathognomonic [**4**], the preoperative diagnosis is rarely established. Interestingly, in a study of 600 cases, Yamaguchi et al. reported a preoperative diagnostic rate of only 5.7% [**8**]. The most frequent initial diagnosis has been reported to be acute appendicitis [**6**]. This happens since as in our case, right lower quadrant abdominal pain is usually attributed to appendiceal inflammation [**6**]. Technetium-99m-pertechnetate scintigraphy is considered the most useful non-invasive diagnostic procedure in the pediatric population, for the detection of ectopic gastric mucosa [**7**], while laparoscopy is a useful diagnostic modality in ambiguous cases [**7**]. 

 Management of complicated Meckel’s diverticulum consists of diverticulectomy in the majority of cases [**7**] along with antibiotics and peritoneal irrigation in case of localized or generalized peritonitis [**5**]. Early intervention is crucial especially when the patient’s state deteriorates [**9**]. Controversy exists regarding the management of asymptomatic Meckel’s diverticulum incidentally discovered during surgical operation [**7**]. Some physicians are supporters of a conservative attitude while others support prophylactic resection in order to minimize the risk of complications [**7**]. In this direction a number of criteria have been established in favor of diverticulectomy such as age (<40 years old), gender (males) the size of the diverticulum (> 2cm) and the presence of adhesions [**10**]. 

 In 1933, Dr Charles Mayo stated that ‘Meckel’s diverticulum is frequently suspected, often overlooked and seldom found’ [**1**]. It usually represents an incidental finding during operation for other abdominal pathology. Symptomatic Meckel’s diverticulum is rare and frequently misdiagnosed, even in developed countries [**1**]. For this reason, the condition should be considered in the differential diagnosis of right lower quadrant abdominal pain and positive history of a swallowed foreign body. Furthermore, surgeons have to maintain a high level of awareness for the morphological and clinical characteristics of this important anatomical structure in order to appropriately manage its complications. 

 Consent 

 Written informed consent was obtained from the patient’s legal guardian for publication of this case report and any accompanying images. A copy of the written consent is available for review by the Editor-in-Chief of this journal. 

 Competing interests 

 The authors declare that they have no competing interests.
